# Nutritional outcomes of empowerment and market integration for women in rural India

**DOI:** 10.1007/s12571-019-00978-z

**Published:** 2019-10-21

**Authors:** Soumya Gupta, Vidya Vemireddy, Prabhu L. Pingali

**Affiliations:** 1 Tata- Cornell Institute for Agriculture and Nutrition, Cornell University, Ithaca, NY USA; 2 Indian Institute of Management Ahmedabad, Ahmedabad, India; 3 Charles H. Dyson School of Applied Economics & Management, Cornell University, Ithaca, NY USA

**Keywords:** Women empowerment, Market integration, Gender, India, Nutrition

## Abstract

Over half of all women of reproductive age are affected by anaemia in India. In this paper we study the role that both household market integration and women’s empowerment in agriculture can play in determining women’s dietary diversity. Our analysis is based on primary data from 3600 households across India on agriculture, nutrition and anthropometric outcomes. We account for market integration by way of per capita household purchases (quantity) of cereals and non- cereal food groups, such as pulses, meat/ fish/ poultry, fruits and vegetables, eggs and dairy. We construct an adapted version of the Abbreviated Women’s Empowerment in Agriculture Index (A-WEAI) that is context- specific and agriculture- oriented. After controlling for individual, household and village- level explanatory factors, we find that – for a given level of per capita market purchases – women who are empowered in their agricultural decisions have significantly higher dietary diversity scores relative to women who are disempowered of such decisions. More specifically it is women’s empowerment in two areas: input in production decisions and membership in self- help groups that supports this result. Women’s empowerment also enhances dietary diversity in the presence of disaggregated per capita purchases of non-cereals such as pulses, meat, dairy and eggs. This highlights the importance of reorienting India’s agricultural price and procurement policies beyond staple grains to ensure better dietary diversity.

## Introduction

The rural poor are among the most food insecure and malnourished people in the world, and are also most often directly involved in small-scale agricultural production. Amongst these, women (especially of childbearing age) are more likely than men to be involved in agricultural activities and are more vulnerable to malnourishment (Herforth et al. 2012). According to the FAO (FAO 2011) women comprise just over 40% of the agricultural labour force in the developing world and about 35% in South Asia. They are involved in production of crops, care of livestock, preparation of food as well as in activities related to trade and marketing of produce. In India the female share of agricultural labour force has remained steady at around 30% from 1980 to 2010, with the sector being a greater source of employment for women relative to men (FAO 2011 WP). A look at women’s time use in agriculture indicates that women in agriculture contribute about 32% of the time required for all agricultural activities in India (FAO 2011 WP). While women form an important part of the agricultural labour force, they are often characterized by a poor nutritional status. India has the largest number of anaemic women followed by China, Pakistan, Nigeria and Indonesia. Over half of all women of reproductive age are affected by anaemia in India (Global Nutrition Report 2017).

Two strategies that have been promoted to improve the nutritional outcomes for women and their households are market integration and women’s empowerment. For rural households, agricultural markets are an important source for households to access ‘income, assets, and factors of production and consumption’ (World Bank 2009). Pingali and Sunder (Pingali and Sunder 2017) highlight the role of markets in nutrition- sensitive food systems by bringing an economic lens to the traditional agriculture- nutrition pathways. As countries go through the process of structural transformation, the decoupling of production and consumption decisions is accompanied by a simultaneous change in demand for food that shifts from staples to non- staples such as fruits, vegetables, meat and dairy products. In this context, well- established and well- functional food supply chains can ensure “availability, affordability, diversity and nutritional quality of foods” (FAO 2013). At the same time, women’s empowerment in agriculture can hold potential for improving nutritional outcomes at the individual and household level. Women’s inputs in production decisions, their control over income and time- use, are all factors that can influence their own nutritional outcomes as well as those of other members of their household. The evidence base for both these pathways (market integration and women’s empowerment) has been explored in different countries at different times, and is mixed at best (discussed in detail in the following section).

In the next section we present the current status of the literature on women’s empowerment and market integration as distinct pathways for improving women’s nutrition and use that to motivate our interest in accounting for the role of both pathways together.

### The empowerment of women and market integration as distinct agriculture- nutrition pathways

Both women’s empowerment and market integration have been proposed as two distinct pathways in the literature on agriculture- nutrition linkages. We discuss each of these briefly in this section.

The women’s empowerment pathway assumes that women can influence household food consumption as both food producers and consumers. As food producers, women’s input in which crops to cultivate and how much of which crops to sell can influence the nutrient- mix of food from own- production, as well as the income for subsequent food purchases. Women’s control over income and input in decisions related to purchase of food can influence the diversity of food basket that is purchased from local market. Further, women’s time allocation between farm work and other non- agricultural chores can determine the amount of time they can devote to food preparation and thereby indirectly influence the choice of foods that are prepared at any given time. Such aspects of women’s decision- making and control over resources are incorporated in the Women’s Empowerment in Agriculture Index (WEAI) which was introduced in 2012 (Alkire et al. 2013). The WEAI is a multi- dimensional index that captures women’s agency in five domains of agriculture – production, resources, income, leadership and time use. The WEAI has recently gained traction in determining the relationship between the empowerment of women in agriculture and their nutritional outcomes. That relationship has varied across countries and across target groups (households, women and children). The aggregate WEAI score has been positively associated with dietary diversity at the household level in Bangladesh (Sraboni et al. 2014). At the individual level, the aggregate empowerment score (Malapit et al. 2015) and its sub-indicators (Ross et al. 2015) have been associated with diet diversity in Ghana. While the WEAI has been associated with the production orientation of households in India (Gupta et al. 2017), there is, to our knowledge, no paper that accounts for how the empowerment of women can influence the quality of market purchases of foods for improved dietary intake.

The second agriculture pathway that is relevant for this research is the role that markets can play as a source of income. Different analyses of crop sales have shown that not only do the majority of smallholder farmers engage in crop sales, but also that most of those households are producing and selling food crops such as staples, vegetables, nuts and seeds (Carletto et al. 2017; Jones 2017). However while agricultural commercialization has been promoted as a strategy for improving nutritional outcomes for smallholder farming households, the evidence base for this is mixed at best, as summarized by Carletto et al. (2017) for various countries. More recent studies have reoriented the debate on the role that markets can play in terms of integration, relative to production diversity, for improved nutritional outcomes. Work done by Sibhatu et al. (2015) in Indonesia, Kenya, Ethiopia and Malawi, and by Koppmair et al. (2017) in Malawi show a significant association between various measures of market integration and dietary diversity. On the other hand, Carletto et al. (2017) find no significant relationship between agricultural commercialization on both child anthropometric outcomes and household calorie intake. These studies emphasize the role of markets as a source of income for producers. This is apparent in the use of indicators such as the area under cultivation of non- food crops (Koppmair et al) and the fraction of harvests sold (Koppmair et al. 2017, Jones 2017, Carletto et al. 2017). However, increased income from increased commercialization is not a sufficient condition for the purchase of a more diverse basket of foods from local markets. For instance Jones (2017) finds that while households with a greater proportion of harvest sold see a higher income from such sales in Malawi, the proportion of purchased foods that they consume, and the average number of food groups they spend on, are not different from households that are less integrated with markets. Given that smallholder farming households rely on markets as source of food (Jones 2017) the treatment of markets in a way that reflects purchase behaviour of households is lacking in recent studies. For this reason, the present study focuses on markets as a source of nutritious foods for the poor. In our analysis, we use quantities of foods purchased by the household for six different food groups as indicators of a household’s market integration. Further, we bring an empowerment- lens to the relationship between market integration and nutrition by hypothesizing that for the same level of market purchases, empowered women are likely to have a higher dietary diversity relative to disempowered women.

Two recent analyses consider empowerment and market integration simultaneously (Fischer and Main 2012, Njuki et al. 2011). Both analyses consider the gendered effects on control over income (proxy for empowerment) from smallholder market integration. Fischer and Qaim (Fischer and Qaim 2012) find that being a member of a farmers’ group increases men’s control over production of and revenues from sale of bananas in Kenya, while Njuki et al. (2011) find that the extent to which certain crop- linked incomes are controlled by men (vs women) depends on the type of commodity (i.e. crops that are or are not traditionally ‘women’s crops’) in Malawi and Uganda. Both studies do expand the analysis to nutritional outcomes although in different ways. Fischer and Main (2012) find that household dietary quality is positively and negatively associated with income from sale of bananas through a farmers group, and men’s control over those revenues respectively in Kenya. While this takes into account the gendered aspect of collective action platforms in determining control over income it does not specifically focus on the empowerment of women in various domains of agriculture and their own nutritional outcomes. Moreover, the paper stops short of analysing the relationship of women’s group membership with household nutritional outcomes. Rather, the analysis reflects that women’s participation in farmers group can have positive implications for women to generate and have control over income. Njuki et al. (2011) find that there are significant differences in the proportion of income spent on food by men and women. However, this is based on group-wise t-tests and does not account for the role of other explanatory variables. In general, there is therefore a lack of studies that account for the empowerment of women in agriculture together with the household’s reliance on markets for food consumption as determinants of women’s nutritional outcomes. Moreover, recent studies have focused on Sub-Saharan Africa as opposed to South Asia, the regional focus of this paper. Market integration in South Asia is much higher as compared to Sub-Saharan Africa (Reardon et al. 2009)

### Objectives

The objective of this paper is to analyse the relationship between market purchases, women’s empowerment in agriculture and their dietary diversity. Our analysis is based on rich primary data collected from 3600 households across four districts in India on various aspects of agriculture, empowerment, WASH, seasonal food deficits and demand for nutritious foods as well as anthropometry for women. The empowerment of women is conceptualized in terms of the Abbreviated WEAI (AWEAI) that is computed using a sharper dataset for women’s participation and decision- making in agriculture in India, based on our field experience. Market integration is estimated using data on purchase of cereal and non- cereal food groups. We argue that both the empowerment of women and market integration can be thought of as a complementary strategy for addressing poor nutrition outcomes in women. For the same level of market integration, we expect empowered women to have better nutritional outcomes, relative to women who are disempowered. Empowered women are better able to translate household market purchases to individual diet improvements.

The rest of this paper is structured as follows. Section 2 discusses methods related to data collection, construction of variables and data analysis. Section 3 presents results and section 4 concludes with recommendations.

## Methods

### Sites and data

**Fig. 1:**
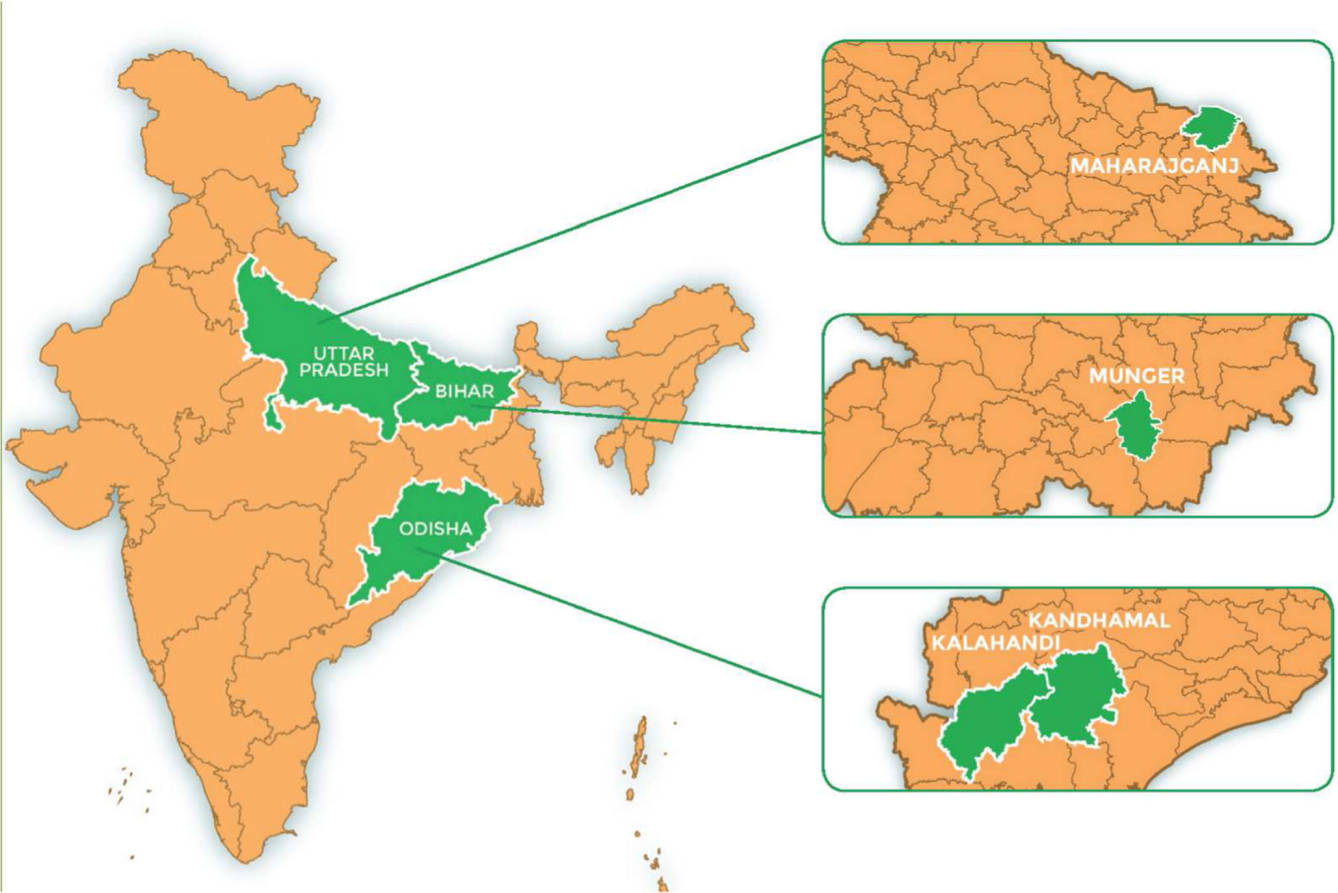
Field- locations: States and Districts

We use data from a primary survey that was conducted in March–May 2017 as part of the Technical Assistance and Research for Indian Nutrition and Agriculture (TARINA) program in India. The TARINA program, led by the Tata-Cornell Institute for Agriculture and Nutrition (TCI) at Cornell University is a consortium of research and development organizations working on the design and promotion of nutrition-sensitive food systems in India. The TARINA baseline survey was administered to a total of 3600 women in four districts that are spread across the states of Uttar Pradesh, Bihar and Orissa. The districts are: Munger (Bihar), Maharajganj (Uttar Pradesh or UP), Kandhamal (Odisha) and Kalahandi (Odisha) – see Fig. 1. Across all these districts, about 20–30% of the labour force was involved in agricultural activities. About 1/4th of them who are involved in these activities are farmers and the rest are agricultural labourers. This pattern prevails across all the districts in our study. About 80–88% of the cultivable area is intended for staple grains such as rice and wheat and the rest is for cultivating pulses. Maharajganj (358 10^3^ ha) and Kalahandi (275 10^3^ha) have the largest area under cultivation, followed by Munger (57 10^3^ha) and Kandhamal (50 10^3^ha). There are differences across these districts in terms access to electricity, water and sanitation. According to the 2015–16 National Family Health Survey (NFHS), more than 85% people practice open defecation in the districts of Odisha (Kalahandi and Kandhamal), whereas the levels are about 25–30% in Maharajganj and Munger. Access to electricity is highest in Maharajganj (73.5%), followed by Munger (50.5%). The same is not true for the districts in Odisha where the households with electricity access are about 38–40%. As regards women’s nutritional status, the National Family Health Survey (IIPS and ICF 2017) data indicates that on average less than 30% of women consumed dairy products, green leafy vegetables and fruits across the four surveyed districts. Further, less than half the women reported having consumed meat/ fish/ poultry or eggs in the previous 24-h. About 35–37% women are underweight in Maharajganj and Kalahandi as compared to Kandhamal (30%) and Munger (32%). As regards women’s BMI levels, the proportion of women who are overweight/obsese is highest for Maharajganj (15%), followed by Munger (12%), Kalahandi (7%) and Kandhamal (6%) (NFHS 2015–16).

The survey was designed using a two-stage sampling strategy. In the first stage, a total of 30 villages per district were selected based on population size and areas of implementation of the TARINA field-level partners. In the second stage, 30 households from each village were selected randomly. This resulted in a total sample of 3600 households across 120 villages.

### Compliance with ethical standards

This research was carried out by the Tata-Cornell Institute for Agriculture and Nutrition (TCI) at Cornell University and was approved by Cornell’s Institutional Review Board. Informed consent was recorded verbally from all individual participants included in this study. Verbal consent was chosen over written consent, as the survey was computer-based. Responses to the informed consent process were recorded accordingly in the survey software.

### Measurement of market integration

We measure market integration in terms of the quantity of per capita food purchases. We focus on the quantities (in kg or units) purchased of the following six food groups: 1) cereals, 2) pulses, 3) vegetables and fruits, 4) eggs, 5) dairy, and 6) meat/ fish poultry (MFP). This enables us to focus not just on cereals, which form the food base in India, but also on foods that are rich in protein and micronutrients and often lacking in diets. Women were asked if the household purchased food items belonging to any of these categories in either the past 7 days or the past 30 days. The reference periods for household purchases of fruits and vegetables and for eggs, dairy, meat and fish are the past 7 days and the past month, respectively. These reference periods are meant to capture the seasonal and perishable nature of food and differences in frequency of consumption of different foods. We derive the per capita purchase values by dividing the household market purchases (both total and by food-group) by household size.

### Measurement of women’s empowerment in agriculture

A revised version of the Abbreviated Women’s Empowerment in Agriculture Index (AWEAI) was developed for the purpose of the present study. The A-WEAI consists six sub-indicators that measure the empowerment of women in five domains of agriculture (Malapit et al. 2015). For the purpose of our analysis we use five of the six sub-indicators that constitute the AWEAI. We replace the workload sub-indicator with leisure. The five domains and constituent sub-indicators and activities are described in Table 1. Appendix 1 presents the differences between the construction of the AWEAI and the index calculated here in detail.

**Table 1:** Components of the revised AWEAI used in this study

Domain name	Subindicator	Explanation of subindicator	Weight
Production	Input in production decisions	A woman is considered adequate in this domain if she has at least some input in two activities: i) which crops to plant ii) technology to adopt iii) sale of crops in market Buy/sell livestock iv) buy/Sell KG produce v) collection of forest produce.	1/5
Resources	Ownership of assets	A woman is considered adequate if she owns any agricultural land solely/ jointly.	1/10
	Decisions on credit	If a woman takes agricultural credit and has input in the decisions in the use of that credit, she is considered adequate in this domain.	1/10
Control over income	Control over income	A woman is considered adequate if there is at least one activity in which she has input in controlling income: i) Income from sale of crops ii) Income from sale of livestock iii) Income from collection of forest produce iv) Income from ag-daily labour.	1/5
Group membership	Self- Help Group (SHG) membership	A woman is considered adequate in this domain if she is an SHG member and she joined the SHG for i) doing collective livelihood or ii) receiving free seeds and samplings for homestead gardens or iii) access to subsidized custom hiring of implements for agricultural activities iv) information about health, nutrition, education and WASH or received training for agriculture activities, livestock activities and kitchen garden activities.	1/5
Workload	Leisure	Adequacy is defined if a woman feels she is satisfied with her free time	1/5

Each of the five domains is equally weighted as 1/5 to compute the empowerment index, which is calculated as:

$$ {\displaystyle \begin{array}{c}{e}_i(k)=1\  if\ {c}_i\ge 0.4\  and\ {e}_i(k)=0\  if\ {c}_i<0.4, where\ k\  is\ the\ cutoff(0.4),\\ {} where\ {c}_i={\sum}_j{w}_j{c}_j\\ {}\begin{array}{c}{c}_j\  is\ adequacy\ in\ each\ subindicator\ and\ the\ {w}_j\  is\ the\ corresponding\ weight;\\ {}i\  is\ the\ in dividual\ and\ j\  represnts\ the\  sub- indicator\end{array}\end{array}} $$

First we generate the adequacy scores for each individual woman. This adequacy is constructed by summing the weighted adequacies across all the sub-indicators (*c*_*i*_). This adequacy score ranges from 0 to 1, where 0 means that adequacy is not achieved in even one domain and 1 is interpreted as adequate in all domains. A higher score is representative of a greater number of domains in which a woman has attained adequacy. We consider a woman “empowered” *e*_*i*_(*k*) when adequacy (*c*_*i*_) is achieved in at least two domains (greater than an adequacy score of 0.4). Accordingly, an individual woman is given a score of 1 (if empowered) and 0 (if disempowered).

### Measurement of women’s dietary diversity

A Dietary Diversity Score (DDS) was constructed for each woman based on the number of food groups she consumed over the previous 24-h. We use the 10-food group classification recommended by the FAO for the Minimum Dietary Diversity for Women indicator (U. & F. FAO 2016). These ten food groups are cereals, pulses, green leafy vegetables, Vitamin A rich fruits and vegetables, other fruits, other vegetables, eggs, meat/ fish/ poultry, dairy, and nuts and seeds. Food items within each of these food groups were identified through a process of community focus group discussions, key informant interviews and secondary data on commonly consumed foods in the four districts. A woman is assigned a score of 1(or 0) for every food group she reports having consumed (or not) in the last 24-h. The DDS reflects increasing dietary diversity as the score increases from 0 to 10. The individual DDS has been validated to reflect dietary quality. A 24-h recall enables us to understand a short-term/ recent snapshot of dietary intake.

### Measurement of productivity diversity

Production diversity has commonly been identified as a significant determinant of dietary diversity in recent literature to varying degrees (Jones et al. 2014; Bhagowalia et al. 2012; Kavitha et al. 2016). It is most often conceptualized as a simple count of the number of crops cultivated (also known as species richness) and sometimes as a count of crop and livestock species. For the purpose of this paper we define the metric based as a simple count of the number of non- staple food *groups* being cultivated in three different seasons. The non-staple food groups we focus on are pulses, oilseeds and nuts, vegetables, and green leafy vegetables. Each non-staple food group gets a score of 1 if there is at least one constituent crop/ pulse/ vegetable that is cultivated by the household in a given cultivation year. The production diversity score therefore can range from 0 to 4. We exclude the cereals food group since staples like rice and wheat are cultivated by majority of the households in our field locations. In that context, by focusing on the non-staples we are better able to highlight the differences in the diversity of agricultural production between households across locations.

### Empirical specification

We analyse the relationship between market integration, women’s empowerment and nutritional outcomes using a multivariate regression model. The outcome of interest is the dietary diversity scores of women. Since the dietary diversity score is a count variable (it ranges from 0 to 10) we estimate Poisson regression models with robust standard errors clustered at the village level. The Jarque-Bera normality test was conducted to determine that the distribution was non-normal.

First, we present the relationship of interest by considering the total per capita market purchases for each woman *i* (*TMKTIT*_*i*_) together with the empowerment status of each woman (*EMP*_*i*_ in eqs. 1 and 2). *EMP*_*i*_ in each equation refers to the woman’s empowerment status (binary). Equation 1 also accounts for interaction effects on dietary diversity scores of women by including the interaction term: *EMP*_*i*_ ∗ *TMKTIT*_*i*_ . In equation 2, the per capita market purchases are decomposed into their constituent food groups, as reflected in the vector *MKTINT*_*ij*_ . This vector has all the per capita quantities of six food groups (*j = 1…6*) purchased by the household – cereals, pulses, fruits and vegetables, dairy, eggs, and meat/ fish/ poultry.

1 $$ Dietary\ {Diversity\ Score}_i={\beta}_0+{\beta}_1{TMKTIT}_i+{\beta}_2{EMP}_i+{\beta}_3{EMP}_i\ast {TMKTIT}_i+{\beta}_3{HH}_i+{\beta}_3{VILL}_i+{\varepsilon}_i $$

2 $$ Dietary\ {Diversity\ Score}_i={\beta}_0+{\beta}_{1j}{MKTINT}_{ij}+{\beta}_2{EMP}_i+{\beta}_3{HH}_i+{\beta}_3{VILL}_i+{\varepsilon}_i $$

In the above equations we control for household-level factors that can influence dietary diversity. *HH* is a vector of household-level control variables: production diversity of non-staples, and the value of sales of cereals (in thousand Rupees as a proxy for income). We also account for the household caste (Scheduled Caste or SC/ Scheduled Tribe or ST, Other Backward Class or OBC or others). *VILL* is the vector of village-level control variables. At the village-level we account for whether or not there is a ration shop as part of the Public Distribution System (PDS) store within 5 km. We include a dummy district variable in all the regressions to control for any district level variation.

In a third specification the disaggregated empowerment index is combined with the total per capita quantity of purchases. *SUBINDICATOR*_*ij*_ is the vector of all the sub-indicators *j* of the WEAI for each woman *i*. This allows expanding the association between market purchases and empowerment to specific domains of empowerment.

3 $$ Dietary\ {Diversity\ Score}_i={\beta}_0+{\beta}_1{TMKTIT}_i+{\beta}_{2j}{SUBINDICATOR}_{ij}+{\beta}_3{HH}_i+{\beta}_3{Vil}_i+{\varepsilon}_i $$

## Results

### Descriptive statistics

Table 2 presents descriptive statistics for the four field locations considered. We disaggregate the averages for outcome, independent and control variables across the four districts. Across districts, women consumed 4 food groups out of 10 on average in the previous 24 h. The per capita quantities purchased are highest for cereals, relative to protein and micronutrient-rich food groups. The per capita quantities purchased are lowest for meats, eggs and dairy products. Between districts, the market purchases of pulses, vegetables, eggs, dairy as well as meat and fish are highest in Munger. Around 60% women in Munger, Maharajganj and Kandhamal are empowered in agriculture. This is about 15 percentage points higher than in Kalahandi. The average number of crops produced by households (production diversity) is highest in Maharajganj, followed by Munger and then the two districts of Odisha. The value of sale of cereals is highest in Kalahandi at INR 8800 on average. It is one-half of that in Munger and one-fifth of that in Maharajganj and Kandhamal. Household production diversity, based on the number of non-staple food groups produced, is 1 for less than 15% of the households in Munger and Maharajganj, and even lower for Odisha. Majority of the households across districts are able to access the PDS at a distance of 5 km or less from their village. The dominant caste groups are Scheduled Caste or SC, Scheduled Tribe or ST and Other Backward Class or OBC in Odisha and Uttar Pradesh respectively.

**Table 2:** Descriptive statistics

	Munger		Maharajganj		Kandhamal		Kalahandi		Total	
	N	Mean	N	Mean	N	Mean	N	Mean	N	Mean
Outcome variables										
Dietary diversity score (Range:0–10)	900	3.87	900	3.76	899	4.76	899	4.73	3598	4.28
Independent variables										
Per capita market purchases of cereals (kg)	484	5.26	462	4.53	610	4.19	558	3.61	2114	4.40
Per capita market purchases of pulses (kg)	588	0.69	859	0.87	858	0.91	772	0.93	3077	.85
Per capita market purchases of fruits and vegetables(kg)	846	1.07	859	0.99	862	1.01	867	1.06	3434	1.03
Per capita market purchase of meat and fish (kg)	330	0.17	336	0.12	502	0.22	541	0.26	1709	.19
Per capita market purchase of eggs (no.)	107	0.29	287	0.34	492	0.69	441	0.71	1327	.51
Per capita market purchase of dairy (kg)	305	0.43	256	0.21	177	0.09	125	0.08	863	.20
Empowerment status of women (Range: 0–1)	900	63%	900	61%	900	62%	900	47%	3600	61%
Control variables										
Value of sale of cereals (in ‘000 rupees)	566	4.82	739	1.29	525	1.3	640	8.87	2470	4.06
Household Size	900	5.08	900	5.36	900	4.55	900	4.43	3600	4.85
Production Diversity of non-staples	900	13%	900	12%	900	3%	900	10%	3600	21%
Presence of PDS^1^ within 5 km from the village (Y/N)	900	0.8	900	0.97	900	0.87	900	0.57	3600	1.2
Proportion of households by market distance										
Within the village	26.67 40 16.67 16.67	20 26.67 46.67 6.67		0 6.44 36.33 57.22		30 10 23.33 36.67		19.17 20.78 30.75 29.31		
Within 2 km from the village										
2-5Km from the village										
Above 5 km										
Caste										
OBC^2^	44.08 40.02 15.89	75.89 24.11		12.36 87.64		28.44 71.56		40.21 55.92 3.87		
SC/ST^3^										
Others										

Note: 1. PDS refers to an abbreviation of Public Distribution system in India. This is a national level food security scheme by the Government of India. Under this scheme, cereals such as wheat, rice and sugar, salt etc. are distributed monthly to families below the poverty line. 2. Other backward castes, 3. Scheduled castes and Scheduled tribes. (These are various caste denominations for the disadvantaged classes in India)

Table 3 reports the differences in the average dietary diversity scores of women, by their empowerment status. We find that women who are empowered have significantly higher dietary diversity scores than those who are disempowered in all districts except Kandhamal.

**Table 3:** Differences in dietary diversity across all districts by women’s empowerment status

	Not-Empowered	Empowered	Difference	(*p* value)
Munger	3.76	4.25	−0.49	0.00***
Maharajganj	3.94	4.15	−0.21	0.06**
Kandhamal	4.86	5.03	−0.17	0.28
Kalahandi	4.71	5.05	−0.34	0.01***

### Role of markets and women’s empowerment for dietary diversity

**Fig. 2:**
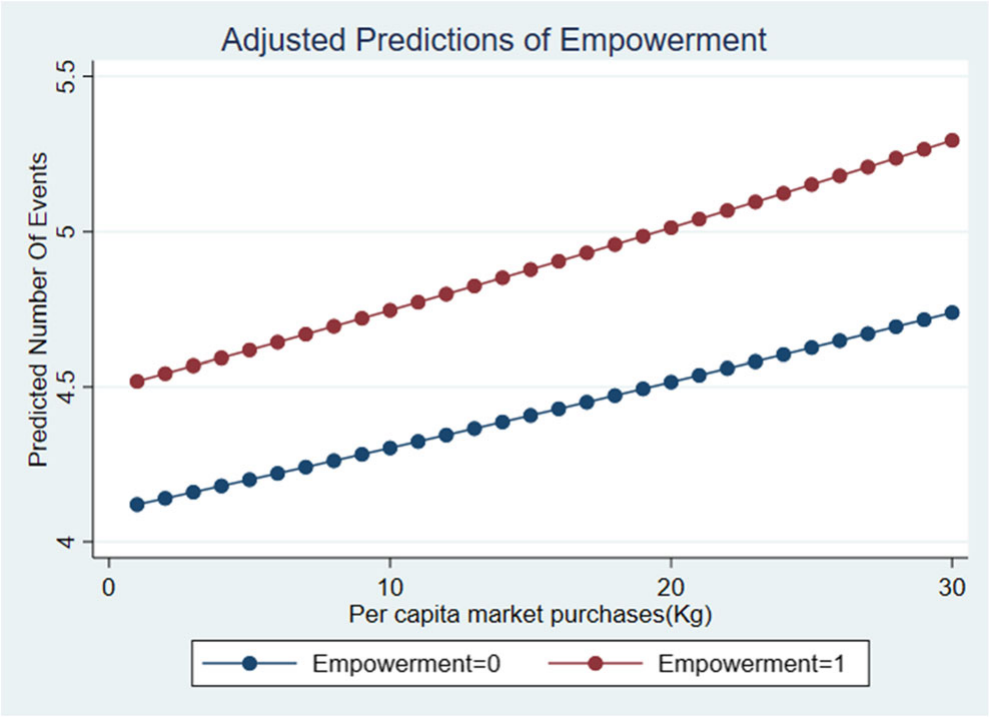
Margins plot for women’s predicted dietary diversity scores by their empowerment status

Table 4 presents results for the role of market purchases and women’s aggregate empowerment status in determining dietary diversity. In specification 1 for a given level of market purchases, women who are empowered have a higher dietary diversity score as compared to women who are not empowered. Taken together these results are also reflected in Fig. 2. Table 3 also indicates that upon disaggregating the per capita total purchases into different food groups, it is the non-cereals, and not cereals, that are significant determinants of dietary diversity (specification 2). In both the specifications an increase in income as measured by the value of sale of cereals has a positive and significant effect on dietary diversity scores by 0.1%. However, the relative importance of market purchases is highlighted in specification 2 where the magnitude of all the market purchases coefficients combined is about 13% higher than the magnitude of empowerment effect and higher than production diversity as well.

**Table 4:** Parameter values estimated in the empirical models for relationship between empowerment, market integration and nutritional outcomes

	Specification 1	Specification 2
	Women’s dietary diversity score	
	*β*/*se*	*β*/*se*)
Empowerment status of the woman (binary)	0.0905*** (0.0246)	0.0846*** (0.0191)
Per capita total market purchases(kg)	0.00469* (0.00188)	
Empowerment status of the woman (binary) = 1 # Per capita market purchases(kg)	0.000470 (0.00257)	
Value of sale of cereals (in ‘000 rupees)	0.00169** (0.000534)	0.00129* (0.000507)
Production diversity of non-staples	0.0283 (0.0257)	0.0286 (0.0243)
Presence of PDS within 5 km from the village	0.0637+ (0.0334)	0.0568+ (0.0344)
SC/ST	−0.0145 (0.0228)	−0.00457 (0.0230)
Others	−0.00949 (0.0648)	−0.0163 (0.0655)
Maharajganj	−0.0248 (0.0307)	−0.00815 (0.0310)
Kandhamal	0.166*** (0.0373)	0.166*** (0.0382)
Kalahandi	0.157*** (0.0339)	0.157*** (0.0353)
Per capita market purchases of cereals(kg)		0.00172 (0.00162)
Per capita market purchases of pulses(kg)		0.0320* (0.0129)
Per capita market purchases of F&V(kg)		−0.00881 (0.0142)
Per capita market purchases of eggs(units)		0.0203* (0.00792)
Per capita market purchases of dairy(kg)		0.0761*** (0.0176)
Per capita market purchases of MFP(kg)		0.0753*** (0.0191)
Constant	1.291*** (0.0395)	1.255*** (0.0405)
Observations	2431	2431

i) Model 1 and 2 are estimated using a poisson model ii) Standard errors in parentheses and are clustered at the village level. ^+^*p* < 0.10, ^*^p < 0.05, ^**^p < 0.01, ^***^p < 0.001 iii) the per capita market purchases do not include eggs as the measurement was in units

Due to the insignificance of the relationship between per capita cereal purchases and dietary diversity, we use per capita purchases of the non-staples to analyse how each of the empowerment sub-indicator relates to market purchases in explaining dietary diversity. These results are presented in Table 5. The per capita market purchases of all the non-staples combined are significant when interacted with leisure and group membership only. Models 1–6 show that women’s dietary diversity scores increase with women’s ability to take decisions related to production and group membership. When all the sub-indicators are combined with market purchases (specification 7), only input in decisions regarding agriculture lead to higher dietary diversity score (Table 5).

**Table 5:** Parameter values estimated in the empirical models for relationship between empowerment, market integration and nutritional outcomes (by empowerment sub-indicator)

	Independent variable across all specification: Women’s Dietary Diversity Specifications						
	1	2	3	4	5	6	7
	*β*/*se*	*β*/*se*	*β*/*se*	*β*/*se*	*β*/*se*	*β*/*se*	*β*/*se*
Per capita market purchases of non-staples(kg)	0.0352*** (0.00776)	0.0333*** (0.00644)	0.0370*** (0.0106)	0.0294** (0.0102)	0.0380*** (0.00597)	−0.0220 (0.0189)	0.0329*** (0.00903)
Input in decisions regarding agriculture = 1	0.0982* (0.0403)						0.0575 (0.0351)
Input in decisions regarding agriculture = 1 # Per capita market purchases of non-staples(kg)	−0.0113 (0.0132)						
Ownership of assets = 1		0.0627 (0.0450)					0.0558 (0.0367)
Ownership of assets = 1 # Per capita market purchases of non-staples(kg)		0.0114 (0.0135)					
Decisions on credit = 1			−0.0214 (0.0475)				−0.0161 (0.0301)
Decisions on credit = 1 Per capita market purchases of non-staples(kg)			−0.0109 (0.0185)				
Control over income = 1				−0.0585 (0.0378)			−0.0143 (0.0384)
Control over income = 1 Per capita market purchases of non-staples(kg)				−0.000635 (0.0128)			
SHG membership = 1					0.119+ (0.0633)		0.0557 (0.0490)
SHG membership = 1 # Per capita market purchases of non-staples(kg)					−0.0599* (0.0262)		
Leisure = 1						−0.0961+ (0.0503)	0.00515 (0.0424)
Leisure = 1 # Per capita market purchases of non-staples(kg)						0.0630** (0.0204)	
Value of sale of cereals (in ‘000 rupees)	0.00183*** (0.000542)	0.00148** (0.000499)	0.00184* (0.000729)	0.00165** (0.000567)	0.00145** (0.000517)	0.00179** (0.000565)	0.00169* (0.000747)
Production diversity of non-staples	0.00935 (0.0333)	0.0303 (0.0256)	0.00605 (0.0347)	0.00940 (0.0337)	0.0293 (0.0256)	0.0283 (0.0255)	0.00636 (0.0345)
Presence of PDS within 5 km from the village	0.0265 (0.0404)	0.0648+ (0.0351)	0.0648 (0.0441)	0.0293 (0.0424)	0.0628+ (0.0351)	0.0614+ (0.0349)	0.0662 (0.0432)
SC/ST	−0.00853 (0.0296)	−0.00499 (0.0240)	0.0179 (0.0368)	−0.0118 (0.0306)	−0.00672 (0.0239)	−0.00657 (0.0235)	0.0171 (0.0354)
Others	0.0158 (0.0681)	−0.0352 (0.0639)	0.0313 (0.0699)	0.0225 (0.0720)	−0.0390 (0.0649)	−0.0417 (0.0652)	0.0366 (0.0690)
Maharajganj	−0.00872 (0.0368)	−0.0251 (0.0327)	−0.0196 (0.0389)	0.00695 (0.0398)	−0.0148 (0.0323)	−0.0224 (0.0332)	−0.0202 (0.0405)
Kandhamal	0.182*** (0.0426)	0.149*** (0.0394)	0.143** (0.0516)	0.186*** (0.0462)	0.160*** (0.0383)	0.158*** (0.0382)	0.137** (0.0501)
Kalahandi	0.141*** (0.0384)	0.124*** (0.0360)	0.143*** (0.0388)	0.144*** (0.0393)	0.141*** (0.0348)	0.135*** (0.0359)	0.137*** (0.0406)
Constant	1.255*** (0.0484)	1.284*** (0.0410)	1.264*** (0.0543)	1.321*** (0.0507)	1.280*** (0.0388)	1.379*** (0.0609)	1.240*** (0.0719)
Observations	1502	2430	1073	1407	2431	2429	1043

Standard errors in parentheses. ^+^*p* < 0.10, ^*^p < 0.05, ^**^p < 0.01, ^***^p < 0.001 i) All models are estimated using Poisson models ii) All standard errors are clustered at the village level. iii) the per capita market purchases do not include eggs as the measurement was in units

## Conclusion and discussion

This study investigates the role that the empowerment of women in agriculture can play in the improvement of the dietary diversity of women. Our results first indicate that market purchases of non-cereals (pulses, dairy, eggs and meat, fish, poultry (MFP) are significant determinants of women’s dietary diversity. Introducing empowerment as a variable in the analysis shows that, for a given level of total market purchases, women who are empowered in agriculture are better able to translate the influence of these purchases into their diets. At a disaggregated level, the empowerment of women enhances the association between the quantities purchased of pulses, eggs, meats and dairy product, and the diversity of their food intake. The purchase of cereals is not significantly associated with nutritional outcomes in our analysis. Upon disaggregating our A-WEAI, we conclude that the domains that can positively influence the dietary diversity of women is women’s input in production decisions, group membership, and their adequate time for leisure. We also find that women’s ability to make decisions in production activities is a significant determinant of the diversity of their diets.

Taken together, these results highlight the nodes at which public policies can intervene to enhance women’s dietary diversity. The significance of purchases of non-cereal food groups highlights the importance of ensuring that households are able to access non-cereal foods at affordable prices through local markets. The ability of smallholder farmers, including women, to integrate in local markets depends on their ability to produce for the market, their ability to access and connect to a market and the stability of the market (Arias et al. 2013). To encourage production of non-cereals for the market, a reorientation of India’s agricultural price and procurement policies will have to take place since they have historically favoured cereals such as rice and wheat. Ensuring market access to inclusive value chains is important from a nutrition and food security point of view (FAO 2017). The ability of both buyers and sellers of non-cereals to access markets will also require investments in rural infrastructure like roads, transport and storage facilities.

At a social level, increasing women’s ability to influence production decisions can have positive implications for their own nutritional outcomes. The unequal access to, and control over, productive resources for women in their agricultural activities is well documented (FAO 2011). Some of the main drivers of the disempowerment of women in agriculture in India have been their inability to make decisions about the use of productive assets, about access to credit, and about their participation in community groups (Gupta et al. 2017). If women were enabled to have input in production decisions, they could influence nutritional outcomes for themselves and their households in three possible pathways. First, women’s input in which crops to grow could presumably shift cropping patterns away from cereals to pulses, fruits and vegetables and contribute to consumption from own-production. Second, women’s input in sale of produce from farming/ livestock/ kitchen garden can generate income from these sales. Culturally, however, women in many rural settings have less access to markets than men and can undertake fewer activities with economic implications than men. For instance in India women’s participation in marketing of agricultural produce is low when compared to activities like transplanting, sowing and harvesting – about 8.5% to 15% across studies (Aggarwal et al. 2013, Upadhyay 2005). Moreover, the ways in which income from commercial agricultural production can influence nutritional outcomes depends, among other things, on women’s control over that income (FAO 2017). Third, women’s input regarding which technologies to adopt may reduce their drudgery in field-level activities and therefore influence time-use in a way that allows for preparation of more nutritious foods (Johnston et al. 2018).

This analysis also makes the following methodological contributions. We adapt the AWEAI using indicators that more sharply reflect the access and decision making aspects in the context of Indian agriculture. We do this by focusing on site-specific production activities, tangible community-specific notions of ownership and agriculture-specific participation in activities, groups and control over income and credit sources. For instance, by accounting for livestock, kitchen garden and forest produce, we account for women’s participation in production decisions that need not necessarily be farm-specific. This is in contrast to what is often seen in Sub-Saharan Africa where there is a clear demarcation of ‘women’s crops’ vs. ‘men’s crops’. Similarly by restricting our focus to ownership of agricultural land we take into account the fact that ‘ownership’ of other assets like livestock, farm equipment and other durables (that are included in the AWEAI) are not relevant for India where these are often considered as household-goods and therefore the definition of ‘ownership’ does not reflect property rights in any tangible way. For the credit sub-indicator we specifically focus on women who participate in SHGs (Self Help Groups) and go on to account for those SHGs that are in one way or another related to agricultural activities. In this respect we sharpen the focus on the role that SHGs as a platform can play for enhancing the empowerment of women in agriculture, as opposed to considering any SHG, or any community group in general. We also account for household’s market integration from the perspective of markets being a source of nutritious food for smallholder farming communities. From the recent literature discussed in section 1.2, we find that the most common measures of household market integration are indicators such as distance to market, proportion of crop sold, area under cultivation of cash crops and food expenditures. We argue that while these reflect the ease/degree to which a household can access a market, they are proxy measures at best when considering the specific role of markets on improved nutrition. While it is plausible that a shorter distance to market, or greater proportion of crop sold can allow a household to access and afford more foods in a local market, whether or not a household actually chooses to purchase more diverse foods cannot be said with certainty. The same holds true for household food expenditures – a higher expenditure need not necessarily imply the purchase of more diverse foods, it could just as well be on the purchase of more cereals. To overcome this limitation our analysis relies on actual quantities of per capita foods purchases as a measure of the extent to which an individual is integrated with local markets. By doing so we are able to get a sense of rural households’ reliance on local markets for non-cereals and thereby highlight the importance of ensuring that markets can meet this demand in the presence of supporting infrastructure, price and procurement policies. In addition to the WEAI and per capita food purchases we also conceptualize production diversity in a manner that accounts for both, local cropping patterns as well as their nutrient content. We argue that the traditional definition of production diversity (a simple count of the number of crops cultivated in multiple seasons) is not an appropriate reflection of the diversity of production. Rather it is a reflection of the cropping intensity. For instance if a household cultivates two types of crops– rice and wheat – in subsequent seasons (or the same) then production diversity based on a count-metric would equal two. In effect, however both rice and wheat correspond to the same food group: cereals. This distinction between individual crops versus the food group(s) cultivated is relevant since the basic premise of using production diversity, as an explanatory variable for dietary diversity is that it can potentially enhance the *diversity* of diets. Going back to the earlier example of cultivation of rice and wheat in subsequent seasons by a household, our definition of production diversity would assign a score of 1 to it since both belong to the same food group which is cereals.

Our analysis is limited in its use of the AWEAI inasmuch as we do not account for women’s time use. Women’s time use, as measured by the WEAI, is not an adequate representation of women’s time use, since it is based on a 24-h recall and captures time spent in agriculture as well as non-agricultural chores/ activities. Our experience with agricultural surveys in India indicates that women’s time in agriculture is likely to vary between different agricultural activities and across different seasons (Johnston et al. 2018). For this reason a 24-h recall as used in the AWEAI offers an incomplete picture of women’s time poverty in agriculture. To account for this variation our survey collected information on time spent by women in each agricultural activity (such as planting/ transplanting, weeding, applying fertilizer) for the main crop cultivated in each season. What we find is that seasonal averages for time use are very low, more so if they are considered for a particular agricultural activity (Table 6). Another limitation of our analysis is that we assume that total purchases of food groups at the household level are equally distributed amongst household members. While the use of per capita purchases allows us to relate them to individual-level dietary diversity, we implicitly assume that per capita purchase reflects per capita consumption.

**Table 6:** Average number of days worked by women in the kharif season (monsoon) by activity (1 Man day = 8 h)

Activity	Munger	Maharajgaj	Kandhamal	Kalahandi
Buying seeds	3.1	1.6	4.2	4.3
Land preparation/Tilling	5.2	1.8	4.1	5.0
Planting	6.0	3.3	5.0	5.0
Transplanting	7.8	3.9	5.1	5.5
Weeding	6.1	3.9	4.7	5.4
Fertilizer Application	3.0	1.3	3.7	4.1
Spraying (pesticides/herbicides)	3.1	1.4	5.2	4.0
Harvesting	8.1	3.9	4.8	5.5
Selling produce in market	3.7	1.4	4.0	5.0

Our work contributes to an understanding of how the empowerment of women and market purchases can influence nutritional outcomes. This is in line with the FAO’s focus on understanding gender dynamics in changing agri- food systems and promoting gender equality in nutrition- sensitive food systems (FAO 2017). The focus on the need to ensure women’s ability to access and take decisions related to economic resources is also highlighted in SDG 5 on gender equality as part of the 2030 Sustainable Development Agenda. The specific focus on ensuring women’s access to markets is also identified as an area of work by the FAO (FAO 2017). This paper is a first step in bringing together two distinct agriculture-nutrition pathways: income and empowerment, in order to recognize the synergies between the two and to highlight how public policies will need to address complementary pathways in order to improve nutritional outcomes for women in smallholder farming communities.

## Appendix 1

Differences in construction of the AWEAI based on the use of a more sharply focused dataset for agriculture and women’s participation in India.

**Table:**

Sub-indicators	AWEAI	AWEAI (Our analysis)	Weights
Input in production decisions	*Has some input or feels can make decision at least two activities* Food crop farming Cash crop farming Livestock raising Fisheries	*Has some input or feels can make decision at least two activities* Which crops to plant Technology to adopt Sale of crops in market Buy/sell livestock Buy/Sell KG produce Collection of forest produce	1/5
Ownership of assets	*Adequate if self/joint owns at least two small assets or if households owns one large asset* Agricultural land, large livestock, small livestock, fishing equipment, farm equipment (mechanized /non-mechanized), non-farm business equipment, house, large consumer durables, small consumer durables, cell phones, non- agricultural land, means of transportation	*Adequate if self/joint ownership of* agriculture land owned by women	1/10
Decisions on credit	*Adequate if self/joint makes decisions regarding credit and has at least one credit* NGO, formal lender, informal lender, friends and relatives, group based MFI, informal group-based	*Adequate if self/joint makes decisions regarding*: Taking loan for agricultural activities	1/10
Control over income	*Adequate if there is at least one domain in which individual has some input or feels can make decisions regarding wage employment and minor household expenditures.* Food crop farming, cash crop farming, livestock raising, non-farm activities, wage and salary employment, minor and major household expenditures fishing	*Adequate if there is at least one domain in which individual has input in controlling income*: Income from sale of crops Income from sale of livestock Income from collection of forest produce Income from ag- daily labour	1/5
Group membership	*Adequate if a women is a member of at least one group* Agricultural/livestock/fisheries producer groups, water users group, forest users group, credit or microfinance group, mutual help/insurance group, Trade and business association group, civic group, other group	*Adequate if a women is an SHG member and has joined SHG for:* Platform for doing collective livelihood OR Free seeds and samplings for homestead gardens OR Subsidized custom hiring of implements for agricultural activities OR Education about health, nutrition, education and WASH OR Received training for agriculture activities, livestock activities and kitchen garden activities.	1/5
Workload	Adequate if time spent in primary activities is less than 10.5 h	Not included because of low seasonal averages.	
Leisure	Adequate if women are satisfied with the time that they get for leisure	Same as AWEAI	1/5
